# Absolute Reliability and Concurrent Validity of Hand-Held Dynamometry in Shoulder Rotator Strength Assessment: Systematic Review and Meta-Analysis

**DOI:** 10.3390/ijerph18179293

**Published:** 2021-09-03

**Authors:** Claudio Chamorro, Miguel Arancibia, Benjamín Trigo, Leónidas Arias-Poblete, Daniel Jerez-Mayorga

**Affiliations:** 1 Faculty of Rehabilitation Sciences, Universidad Andres Bello, Santiago 7591538, Chile; claudio.chamorro@unab.cl (C.C.); migu.aran@hotmail.com (M.A.); Benja_t9821@hotmail.com (B.T.); leonidas.arias@unab.cl (L.A.-P.); 2 Servicio Kinesioterapia Ambulatoria, Clínica Las Condes, Santiago 7591538, Chile

**Keywords:** muscle strength, reliability, rotator cuff

## Abstract

The purpose of this study is to establish the absolute reliability between hand-held dynamometers (HHDs) and concurrent validity between HHDs and isokinetic dynamometers (IDs) in shoulder rotator strength assessment. The Medline, CINAHL, and Central databases were searched for relevant studies up to July 2020. Absolute reliability was determined by test–retest studies presenting standard error of measurement (SEM%) and/or minimal detectable change (MDC%) expressed as percentage of the mean. Studies considering intra-class correlation coefficient (ICC) between IDs and HHDs were considered for concurrent validity. The risk of bias and the methodological quality were evaluated according to COnsensus-based Standards for the selection of health Measurement INstruments (COSMIN). Thirteen studies were included in the meta-analysis. Shoulder internal rotator strength assessment MDC% was 0.78%, 95% confidence interval (CI) −5.21 to 3.66, while shoulder external rotators MDC% was 3.29%, CI −2.69 to 9.27. ICC between devices was 0.94, CI (0.91 to 0.96) for shoulder internal rotators and 0.92, IC (0.88 to 0.97) for shoulder external rotators. Very high correlation was found for shoulder rotator torque assessment between HHDs and IDs. The COSMIN checklist classified the selected studies as adequate and inadequate.

## 1. Introduction

Muscle strength assessment is relevant in patients with shoulder disorders [1,2]. Isokinetic and hand-held dynamometers (HHDs) are useful tools for clinicians to objectively assess muscle strength, quantify the degree of impairment, and evaluate treatment efficacy when performed before and after an intervention [3,4]. Isokinetic dynamometers (IDs) are considered the reference standard in muscle testing. Maximal torque can be generated throughout the entire range of motion, and results are not underestimated if the examiner is unable to oppose enough force to the assessed muscle strength [5]. Although considered to be the gold standard, isokinetic testing is limited in clinical settings because of the high cost and the laboratory setting required. HHDs are considered low cost, with convenient size and easy use of instruments, but their psychometric properties in shoulder rotator strength assessment are not clearly understood. Relevant psychometric properties include absolute reliability and concurrent validity analysis. Absolute reliability determines variations in repeated measurements performed multiple times under similar conditions [6,7], ensuring that changes between measurements are due to differences in performance instead of inconsistency in measuring the capacity of the applied device [8].

The most common methods for a correct analysis are the standard error of measurement (SEM) reliability and minimal detectable change (MDC) for within-subject variation [9]. They are usually expressed as percentage of the mean (SEM% and MDC%) for analysis purposes. Concurrent validity focuses on the extent to which scores of a certain instrument are related to a well-established gold standard obtained at the same point in time [7]. One common method for analyzing concurrent validity is inter-class reliability (ICC). It determines the degree to which individuals maintain the same position in a sample assessed by different instruments. Few systematic reviews have summarized the results of strength assessment in the shoulder joint. Edouard et al. [10] conducted a systematic review to determine the influence of position in shoulder rotator strength assessment with isokinetic dynamometry, Sorensen et al. [11] in measurement properties of isokinetic dynamometry for assessment of shoulder muscle strength and Schrama et al. [1] in intra-examiner reliability in strength assessment in the upper extremity. No systematic review has been published to determine absolute reliability and concurrent validity in shoulder strength assessment. The aim of this systematic review was to (i) determine the absolute reliability between HHDs and (ii) determine the concurrent validity between HHDs and IDs in maximal shoulder internal and external rotator strength assessment.

## 2. Materials and Methods

The reporting of this systematic review is based on the Preferred Reporting Items for Systematic reviews and Meta-Analyses (PRISMA) guidelines [12]. The PRISMA guidelines consist of a 27-item checklist and a four-phase flow diagram.

### 2.1. Search Strategy

A search was conducted for relevant studies in English published from 1990 up to and including July 2020. The CENTRAL Cochrane, MEDLINE, and CINAHL databases were explored, in addition to gray literature. Muscle strength, isokinetic, dynamometry or dynamometer, validity, reliability, gold standard, shoulder joint, and rotator cuff were included as search terms.

### 2.2. Study Selection

The inclusion criteria for studies were (1) asymptomatic participants; (2) shoulder rotator strength of the dominant side of participants assessed using isometric contractions with either an HHD or ID; (3a) absolute reliability, expressed as the SEM% or MDC% for within-subject variability between trials in maximal shoulder rotator strength assessment, and/or (3b) concurrent validity, expressed as ICC with 95% confidence interval (CI); and (4) for absolute reliability studies, strength assessment expressed in kilos or pounds. For concurrent validity studies, strength assessment was expressed in Newton * meter (Nm).

The exclusion criteria were (1) no full text availability, (2) measures of central tendency and dispersion not mentioned, and (3) concurrent validity expressed in Pearson correlation.

### 2.3. Data Extraction

Titles and abstracts were screened by two reviewers (MA and BT). Full-text review based on the inclusion/exclusion criteria was suggested by yes or no criteria. If discrepancies existed between reviewers, the ratings were discussed until consensus. Studies analyzing absolute reliability were included when the method employed was properly described, especially population, number of subjects, dynamometer model, shoulder assessment position, test–retest procedure, interval between the two tests, and a statistical analysis suitable for reliability tests. Studies analyzing concurrent validity were considered when sample characteristics, HHD model, ID model, and assessment position were mentioned.

### 2.4. Quality Assessment Methodology

The “Consensus-based Standards for the selection of health measurement instruments risk of bias checklist” (COSMIN) [13] was used for methodological assessment. Box 7 was used to assess absolute reliability studies and box 8 for concurrent validity studies. The methodological quality of each item in a box was rated as “very good”, “adequate”, “doubtful”, or “inadequate”. The lowest rating in the box was used for determining the overall quality of a measurement property in each study. Following COSMIN, the results of each study were rated as sufficient (+), insufficient (-), or indeterminate (?), according to Terwee et al. [14]. Absolute reliability measurement was rated as sufficient if MDC was less than 15%. Different studies [15,16] recommend that changes between 10% and 15% are clinically relevant. Concurrent validity was considered as sufficient if the obtained ICC was over 0.70 [13].

### 2.5. Data Synthesis and Analysis

Absolute reliability in shoulder rotator strength assessment was analyzed by SEM% and MDC%. In studies where only SEM was reported, MDC was determined as

MDC = SEM * 1.95 * √2 (23,24).

MDC% was calculated as.

MDC% = (MDC * 100)/(mean test and retest).

Upper limit of 95% confidence interval of MDC% (MDC% upper limit) was calculated as MDC% upper limit = ((mean retest-mean test) * 100/(mean test and retest)) + MDC%.

Lower limit of 95% confidence interval of MDC% (MDC% lower limit) was calculated as

MDC% lower limit = ((mean retest-mean test) * 100/(mean test and retest)) − MDC%.

Concurrent validity was analyzed by ICC between HHDs and IDs, considering a two-way random effects model with absolute agreement. Level of agreement between devices was classified according to Munro’s scale, where values of less than 0.25 represented little correlation; 0.26–0.49 low correlation; 0.50–0.69 moderate correlation; 0.70–0.89 high correlation; and over 0.9 very high correlation [17]. Stata Statistical Software: Release 13 (College Station, TX, USA: StataCorp LP software) was used for statistical analysis. 

## 3. Results

### 3.1. Overall Results

**Figure 1:**
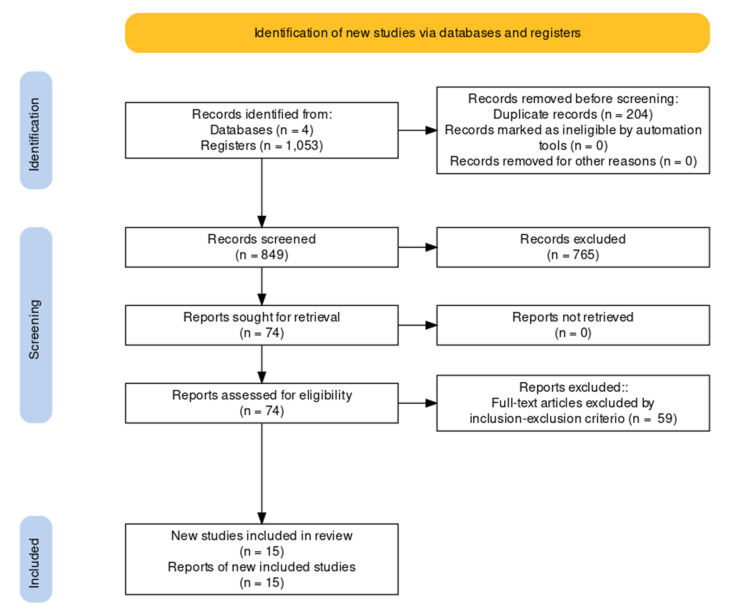
Flow chart of the manuscript selection process.

A total of 1053 studies were selected from the initial search. Seventy-four studies were considered for full-text review after removal of excluded studies and duplicates. Fifteen studies met inclusion and exclusion criteria; 13 of those [18,19,20,21,22,23,24,25,26,27,28,29,30] were considered for meta-analysis. Two studies were not considered due to values not being shown in kgs or pounds for HHD assessments. Ten of the assessed studies [18,19,20,21,22,23,24,25,26,27] provided a detailed review for the MDC% of within-subject variations between trials 1 and 2 for the HHD when assessing muscle strength in shoulder rotators (Figure 1). Descriptions of characteristics and outcomes of studies considering absolute reliability of HHD are presented in Table 1 and Table 2. Descriptions of characteristics and outcomes of studies considering concurrent validity between HHD and gold standard ID are presented in Table 3 and Table 4.

### 3.2. Methodological Quality of Studies

#### 3.2.1. Absolute Reliability

The methodological quality of the selected studies considering absolute reliability is shown in Table 5. One study presented adequate methodological quality [26], while nine studies [18,19,20,21,22,23,24,25,27] were rated as inadequate. Inadequate methodological quality of the studies was mainly due to an inappropriate time interval (fewer than 3 days) and a low sample size (<30).

#### 3.2.2. Concurrent Validity

The methodological quality of the selected studies analyzing concurrent validity is shown in Table 6. One study was scored as adequate [28]. Two studies were scored as inadequate [29,30] due to a small sample size (<30). The ID used in each study was explicitly mentioned and recognized as the gold standard. Independent measurements were assessed between devices, and correlations were calculated in all studies.

#### 3.2.3. Absolute Reliability of HHD and Concurrent Validity between HHDs and IDs Based on the Criteria for Good Measurement Properties

Absolute reliability for shoulder IR strength measurement was rated as sufficient in four studies [18,19,21,24] and as insufficient in three studies [20,22,26]. Absolute reliability for shoulder ER strength assessment was rated as sufficient in four studies [18,19,20,24] and as insufficient in six studies [21,22,23,25,26,27]. Concurrent validity for internal and external shoulder rotator strength assessment was rated as sufficient in all studies [28,29,30] (Table 7).

### 3.3. Meta-Analysis

Thirteen studies [13,14,15,16,17,18,19,20,21,22,23,24,25] were included in the meta-analysis (Figure 1). Ten studies were included for absolute reliability analysis in HHDs [18,19,20,21,22,23,24,25,26,27] and three studies for concurrent validity analysis between HHDs and IDs [28,29,30].

#### 3.3.1. Hand-Held Dynamometry: Absolute Reliability

Effect size is expressed in kgs as percentage of the mean (kg%). One study [19] assessed internal and external rotation strength in two groups (healthy young adults and female athletes) with separate analysis.

##### Shoulder Internal Rotators

**Figure 2:**
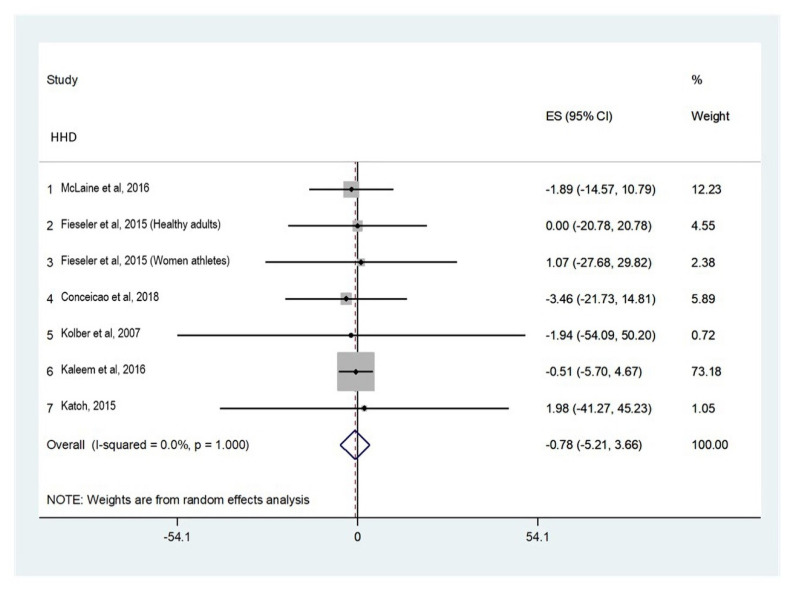
Absolute reliability for shoulder internal rotator strength assessment.

Six studies (Figure 2) were included for shoulder internal rotator strength analysis [18,19,20,21,23,25]. Two of the studies used the Micro FET 2 HHD [20,21]; other HHDs used were Jtech Powertrack, IsoForce Control EVO2, EN-TreeM, and μTas F-1 HHD. Four studies performed the assessment in the sitting position [18,21,23,25], one in the supine position [19], and one in the prone position [20].

##### Shoulder External Rotators

**Figure 3:**
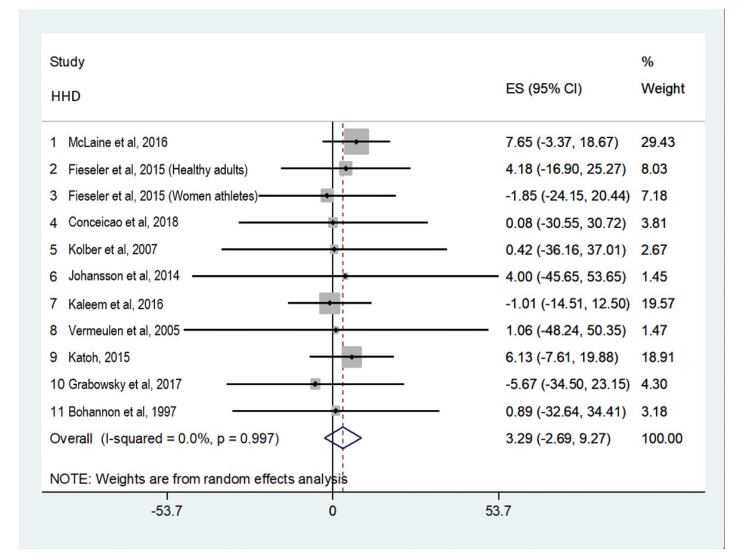
Absolute reliability for shoulder external rotator strength assessment.

Ten studies (Figure 3) were included for shoulder external rotator strength analysis [18,19,20,21,22,23,24,25,26,27]. Three of the studies used the Micro FET 2 HHD [20,21,24]. Six studies performed the assessments in the sitting position [18,21,22,23,24,25], two in the supine position [19,27], one in the standing position [26], and one in the prone position [20].

#### 3.3.2. Concurrent Validity between Hand-Held Dynamometers and Isokinetic Dynamometers

Effect size is expressed as IC and 95% CI for shoulder internal and external strength assessment.

##### Shoulder Internal Rotators

**Figure 4:**
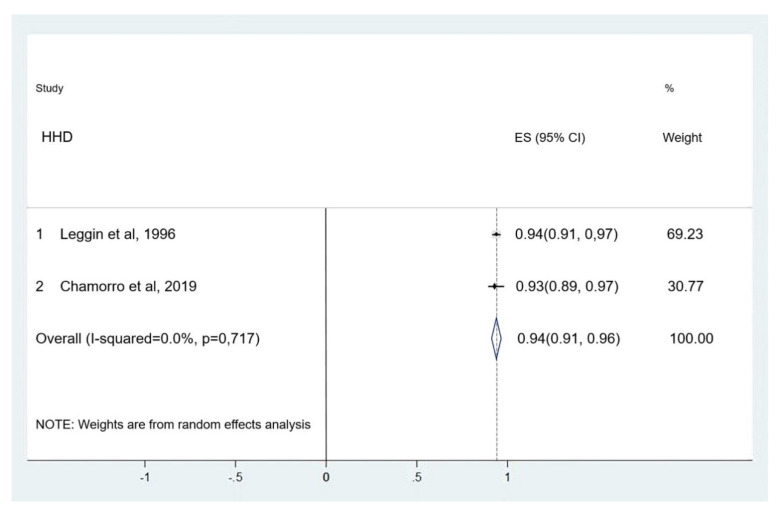
Meta-analysis of concurrent validity for shoulder internal rotator strength assessment.

Two studies were considered for HHD and ID concurrent validity. One study compared the Lafayette HHD to the Biodex ID [28] with the patient seated and shoulder positioned in a scapular plane, while the other study compared the FED HHD to the ID REV 7000 ID device [30] in the supine position and shoulder positioned at 90° abduction (Figure 4).

##### Shoulder External Rotators

**Figure 5:**
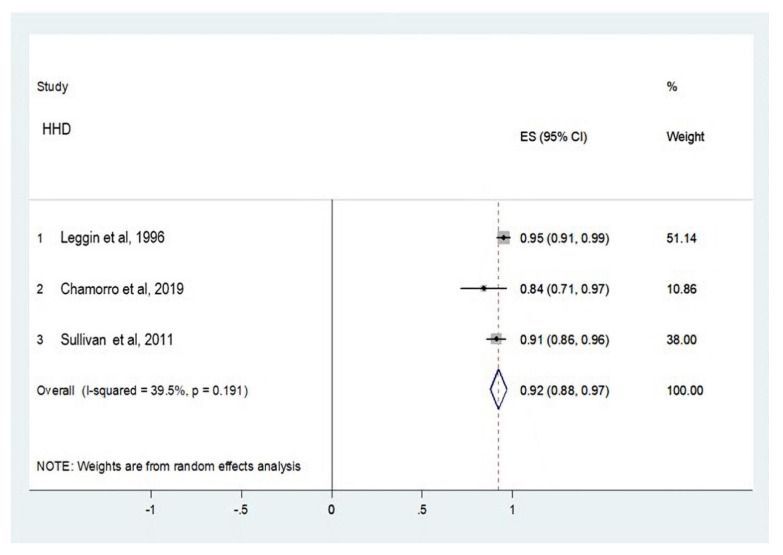
Meta-analysis of concurrent validity for shoulder external rotator strength assessment.

Three studies (Figure 5) were considered for HHD and ID concurrent validity. One study compared the Lafayette HHD to the Biodex ID [28] with the patient seated and shoulder positioned in a scapular plane. The second study compared the FED HHD to the ID REV 7000 ID device [29] in the supine position and shoulder positioned at 90° abduction. The third study compared the Lafayette HHD to the Cybex Norm ID in the supine position and shoulder positioned at 0° abduction [30].

## 4. Discussion

The general results showed good absolute reliability for HHDs in shoulder internal and external rotator strength assessment. Lower and upper limits of MDC% did not exceed 15%, showing low random error of the instrument in strength test–retest evaluation. Minimal clinically important difference (MCID) reflects the smallest measured change in score that patients perceive as important. In strength evaluations, various authors [15,16] argue that the clinical significance is about 15%. In fact, they suggest that differences between the affected and non-affected limbs should not exceed 15% to be able to return safely to sports activity. As this systematic review shows MDC% under the MCID for both internal and external rotation, HHDs can detect small but clinically relevant changes in the assessment of shoulder rotator strength.

The narrower the interval between the upper and lower limits of the MDC%, the greater precision shown by the assessment tool. For example, when analyzing absolute reliability in shoulder external rotator strength assessment (Figure 3 diamond), a confidence interval between -2% and 9% was shown, meaning that if a patient is referred by a physician to an eight-week rehabilitation program, this would be effective for shoulder external rotator strengthening if at least a 9% improvement is observed at the end of those 8 weeks. Only McLaine et al. [18] and Kaleem et al. [23] showed MDC% values under 15% for internal rotation, while for external rotation only Kaleem et al. [23] published lower values. The upper limits of the MDC% in studies that contributed to the review provide a measurement error similar to IDs. This review showed lower MDC% than what was reported by van Meeteren et al. [31], but higher MDC% than reported by Collado-Mateo et al. [32]. The MDC% obtained in the meta-analysis for lower extremities [19] showed higher values than those obtained in this study. This is in accordance with what was stated by Wikholm and Bohannon [33], which establishes that in small groups such as the shoulder rotators, reliability is higher than in larger groups, such as the knee extensors.

Regarding concurrent validity, a very high correlation was established for internal and external rotator strength assessment according to the Munro scale [17]. Concurrent validity between the HHD and the ID was better for internal rotation than for external rotation. Very high correlation between devices and a narrow confidence interval were reported in all studies assessing internal rotation [28,29,30]. Two studies [28,29] classified the concurrent validity between instruments as very high correlation in the assessment of external rotator strength. One study classified it as high correlation. External rotation at 90° or higher glenohumeral abduction generates less activation in coracobraquialis, biceps, anterior braquialis, major pectoralis, and subscapularis, generating less glenohumeral stability. Evidence of anticipated external rotation in shoulder rotators has been published [34]. This could partially explain why reliability and validity are better for internal rotators than external rotators in the shoulder joint.

To the best of our knowledge, this is the first systematic review that summarizes available research on measurement properties in HHDs when used to assess shoulder muscle strength. Although both rotations showed good absolute reliability, better reliability and concurrent validity are observed for the internal rotators than for the external rotators. Several factors with a relevant influence on the calculations of the psychometric properties of the instruments measuring muscle strength must be considered for correct interpretation of the tests.

The stabilization system is related to the external fixation applied to allow the movement to be as analytical as possible and eliminate compensations. For example, when evaluating the shoulder joint, it is suggested to stabilize the pelvic girdle, trunk, and anterior translation of the humeral head. Proximal stabilizer deficiencies affect the distal force evaluated. Incorrect stabilization can also affect the length–tension relationship of the musculature to be evaluated. Unlike the ID, the HHD does not have a stabilization system, so it must be created externally. In all studies, stabilization systems were poorly described.

There are various ways of positioning the subject while assessing the shoulder joint. Articles included seated and supine shoulder evaluations with low and high angles of glenohumeral elevation. It is expected that the supine evaluation can better fix the scapula, which should increase the reliability and the evaluated torque [16]. New studies comparing different subjects’ position for shoulder strength assessment are required.

If the same evaluator carries out the tests, a difference of at least seven days between test and retest is recommended [7]. In many publications, the retest was carried out less than 3 days after the first one, which takes away any independence, since the evaluator remembers the result obtained. Independence in evaluations was achieved only in three studies [19,20,25]. This can overestimate HHDs’ psychometric properties in shoulder rotator strength assessment.

The evaluation protocol must be clearly described so that it can be reproduced. The conditions should be as similar as possible in both the protocol and environmental conditions. For example, if two submaximal tests are performed in a strength test, they must remain constant in the retest [6]. Similar conditions in the test–retest assessment were declared in all studies.

Randomization is one of the ways to reduce random error. If shoulder internal and external rotations are evaluated, there must be a randomization system so that some subjects start with medial rotation and others with lateral rotation. Thus, there is a possibility of systematic errors such as fatigue in lateral rotation due to the same order in performing both types of rotation. As a considerable number of studies show deficits in these relevant factors, the information provided in this review should be interpreted with caution. Future studies with better design in protocols and higher methodological quality are suggested to increase the fidelity of the results.

Publication bias is evident, since only papers published in English were included. Another bias comes from the lack of a universal equation to calculate MDC and ICC. Asymptomatic subjects were considered in this review, so the MDC% values obtained cannot be directly extrapolated to patients with shoulder dysfunction. Finally, concurrent validity of internal rotator strength assessment in this meta-analysis is composed by only two studies and a total sample size of 41 patients, making them less informative than other meta-analyses presented in this study.

## 5. Conclusions

Considering COSMIN classifications, studies were ranked methodologically between adequate and inadequate. Higher MDC% was found for shoulder external rotator strength assessment, although inside the MIC. Very high correlation between devices according to Munro was found for internal and external rotation. Although HHDs appear to be reliable tools for strength assessment in shoulder rotators, results should be considered with caution due to the clinical heterogeneity between studies and some methodological flaws.

## Figures and Tables

**Table 1:** Description of studies analyzing absolute reliability of HHDs.

Authors	Participants	Age(SD)	Type of Dynamometer	Movement	Assessment Position	Shoulder Position	Type of Muscle Contraction	Test–Retest Interval
McLaine et al., 2016	15 healthy adults F:10 M:5	24 (8.2)	Jtech Powertrack Commander	IR/ER	Seated	ABD 90°	Isometric	48 h
Fieseler et al., 2015	25 healthy adults F:13 M:12	21.9 (1.2)	IsoForce Control EVO2	IR/ER	Supine	ABD 90°	Isometric	7 days
	22 female athletes	21 (3.8)						
Conceicao et al., 2018	29 swimmers :21 M:8	16.2 (1.2)	Micro FET 2	IR/ER	Prone	ABD 90°	Isometric	7 days
Kolber et al., 2007	30 healthy adults F:15 M:15	35.6 (13.7)	Micro FET 2	IR/ER	Seated	ABD scapular plane	Isometric	10 min
Johansson et al., 2014	25 healthy adults:16 M:9	25.2 (10)	Compu FET	RE	Seated	ABD 90°	Excentric	20 s
Kaleem et al., 2016	30 male volleyball players	21 (1.8)	EN-TreeM	IR/ER	Seated	ABD scapular plane	Concentric	48 h
Vermeulen et al., 2005	20 healthy adults F:8 M:16	25 (12)	Micro FET 2	RE	Seated	ABD 90°	Isometric	3 days
Katoh, 2015	40 healthy adults H:20 M:20	21(1)	μTas F-1	IR/ER	Seated	ABD scapular plane	Isometric	30 s
Grabowski et al., 2017	44 healthy adults H:24 M:20	21.2 (1.5)	Kiio Sensor	RE	Standing	30° ABD scapular plane	Isometric	7 days
Bohannon et al., 1997	231 healthy adults H:106 M:125	40 (20)	Accuforce II	RE	Supine	45° ABD	Isometric	1 min

F: female; M: male; IR: internal rotators; ER: external rotators; min: minutes; (s): seconds; ABD: abduction.

**Table 2:** Outcome descriptions of studies analyzing absolute reliability of HHDs.

Authors	Movement	Mean (%)	Difference Scores between Trials (%)	SEM (kg) (SEM%)	MDC Lower Limit (%)	MDC Upper Limit (%)
McLaine et al., 2016	IR	10.6	−1.88	0.24 (2.26)	−4.45	8.22
	ER	9.15	7.65	0.18 (1.96)	−13.15	−2.14
Fieseler et al., 2015	IR	9.7	0	0.36 (3.71)	−10.39	10.39
	ER	11.95	4.18	0.45 (3.76)	−14.72	6.35
	IR	9.35	1.06	0.48 (5.13)	−15.44	13.30
	ER	10.8	−1.85	0.43 (3.98)	−9.29	13
Conceicao et al., 2018	IR	15.32	−3.45	0.5 (3.26)	−5.67	12.59
	ER	12.06	0.08	0.66 (5.47)	−15.39	15.23
Kolber et al., 2007	IR	12.35	−1.94	1,15 (9,31)	−24.12	28.01
	ER	9.49	0.42	0.62 (6.53)	−18.71	17.87
Johansson et al., 2014	ER	12.74	4	1.13 (8.86)	−28.82	20.82
Kaleem et al., 2016	IR	19.45	−0.51	0.18 (0.92)	−2.07	3.10
	ER	9.95	−1	0.24 (2.41)	−5.74	7.75
Vermeulen et al., 2005	ER	11.36	1.05	1.06 (9.33)	−27.18	25.07
Katoh, 2015	IR	15.15	1.98	0.96 (6.33)	−19.72	15.76
	ER	8.15	6.13	0.2 (2.45)	−13.00	0.73
Grabowski et al., 2017	ER	9.52	−5.67	0.49 (5.14)	−8.73	20.08
Bohannon et al., 1997	ER	13.53	0.88	0.81 (5.98)	−17.64	15.87

IR: internal rotators; ER: external rotators; SEM: standard error of measurement; MDC: minimal detectable change.

**Table 3:** Description of studies analyzing concurrent validity between HHD and ID.

Authors	Participants	Age (SD)	Movement	Assessment Position	Shoulder Position	Dinamometer	Comparison	Type of Muscular Contraction
Leggin et al., 1996	17 healthy adults F:10 M:7	30.5 (5.5)	IR/ER	Seated	Scapular plane	HHD Lafayette	ID BIODEX	Isometric
Chamorro et al., 2019	24 healthy adults F:19 M:5	23.1 (2.2)	IR/RE	Supine	ABD 90°	HHD FED	ID REV 7000	Isometric
Hebert et al., 2011	74 healthy adults F:36 M:38	10.7 (3.9)	ER	Supine	ABD 0°	HHD Lafayette	ID CYBEX norm	Isometric

F: female; M: male; IR: internal rotators; ER: external rotators; ABD: abduction.

**Table 4:** Outcome descriptions of studies analyzing concurrent validity between HHD and ID.

Authors	Participants	Movement (Strength Measurement Unit)	HHD (SD)	ID (SD)	Interdevice ICC (95% CI)
Leggin et al., 1996	17 healthy adults F:10 M:7	IR (Nm)	NOT SHOWN	NOT SHOWN	0.94 (0.91–0.97)
		ER (Nm)	NOT SHOWN	NOT SHOWN	0.95 (0.91–0.99)
Chamorro et al., 2019	24 healthy adults F:19 M:5	IR (Nm)	32.5 ± 11.1	30.9 ± 13.6	0.93 (0.89–0.97)
		ER (Nm)	23.9 ± 5.81	27.5 ± 12.0	0.84 (0.71–0.97)
Hebert et al., 2011	74 healthy adults	ER (Nm)	NOT SHOWN	NOT SHOWN	0.91 (0.79–0.96)

F: female; M: male; IR: internal rotators; ER: external rotators; ICC: inter-rater correlation coefficient.

**Table 5:** Methodological quality considering the COSMIN checklist for studies included in absolute reliability of HHD (box 7).

Authors	Were Patients Stable in the Interim Period in the Construct to Be Measured?	Was the Time Interval Appropriate?	Were the Test Conditions Similar for the Measurements?	Was the Standard Error of Measurement (SEM), Minimum Detectable Change (MDC), or Limits of Agreement (LOA) Calculated?	Was the Standard Error of Measurement (SEM), Smallest Detectable Change (SDC), or Limits of Agreement (LoA) Calculated?	Were There Any Other Important Flaws in the Design or Statistical Methods of the Study?	Ranking
McLaine et al., 2016	Very good	Inadequate	Very good	Very good	NA	Inadequate	Inadequate
Fieseler et al., 2015	Very good	Very good	Very good	Very good	NA	Inadequate	Inadequate
Conceicao et al., 2018	Very good	Very good	Very good	Very good	NA	Inadequate	Inadequate
Kolber et al., 2007	Very good	Inadequate	Very good	Very good	NA	Adequate	Inadequate
Johansson et al., 2014	Very good	Inadequate	Very good	Very good	NA	Inadequate	Inadequate
Kaleem et al., 2016	Very good	Inadequate	Very good	Very good	NA	Adequate	Inadequate
Vermeulen et al., 2005	Very good	Adequate	Very good	Very good	NA	Inadequate	Inadequate
Katoh, 2015	Very good	Adequate	Very good	Very good	NA	Inadequate	Inadequate
Grabowski et al., 2017	Very good	Adequate	Very good	Very good	NA	Adequate	Adequate
Bohannon et al., 1997	Very good	Inadequate	Very good	Very good	NA	Very good	Inadequate

NA: not applicable.

**Table 6:** Methodological quality considering the COSMIN checklist for studies analyzing concurrent validity between HHD and ID (box 8).

Authors	For Continuous Scores: Were Correlations or the Area under the Receiver Operating Curve Calculated?	For Dichotomous Scores: Were Sensitivity and Specificity Determined?	Were There Any Other Important Flaws in the Design or Statistical Methods of the Study?	Ranking
Leggin et al., 1996	Very good	NA	Adequate	Adequate
Chamorro et al., 2019	Very good	NA	Adequate	Inadequate
Hebert et al., 2011	Very good	NA	Adequate	Inadequate

NA: not applicable.

**Table 7:** Methodology of studies analyzing absolute reliability of HHD and concurrent validity between HHDs and ID based on the criteria for good measurement properties.

Authors	N	Rating MDC for Absolute Reliability HHDs	N	Rating ICC for Concurrent Validity HHD and ID
McLaine et al., 2016	15	MDC IR:8.22 (+) ER:13.15 (+)		
Fieseler et al., 2015	25	MDC IRI:10.39 (+) ER:14.72(+)		
	22	MDC IR:15.44 (-) ER:13.00 (+)		
Conceição et al., 2018	29	MDC IR:12.59 (+) ER:15.39 (-)		
Kolber et al., 2007	30	MDC IR:28.01 (-) ER:18.71 (-)		
Johansson et al., 2014	25	MDC ER:28.82 (-)		
Kaleem et al., 2016	30	MDC IR:3.10 (+) ER:7.75 (+)		
Vermeulen et al., 2005	20	MDC ER:27.18 (-)		
Katoh, 2015	40	MDC IR:19.72 (-) ER:13.00 (+)		
Grabowski et al., 2017	44	MDC ER:20.08 (-)		
Bohannon et al., 1997	231	MDC ER:17.64 (-)		
Leggin et al., 1996			17	ICC IR:0.94 (+) ER:0.95 (+)
Chamorro et al., 2019			24	ICC IR:0.93 (+) ER:0.84 (+)
Hebert et al., 2011			74	ICC ER:0.91 (+)

IR: internal rotators; ER: external rotators; MDC: minimal detectable change; ICC: inter-rater correlation coefficient; sufficient: (+); insufficient: (-).

## Data Availability

Not applicable.
